# Premature mortality in children aged 6–9 years with neurological impairments in rural Kenya: a cohort study

**DOI:** 10.1016/S2214-109X(19)30425-5

**Published:** 2019-10-22

**Authors:** Jonathan A Abuga, Symon M Kariuki, Samson M Kinyanjui, Michaël Boele Van Hensbroek, Charles RJC Newton

**Affiliations:** a Clinical Research (Neurosciences), KEMRI-Wellcome Trust Research Programme, Kilifi, Kenya; b Global Child Health Group, Emma Children's Hospital, Academic Medical Centre, University of Amsterdam, Netherlands; c Department of Public Health, Kisii University, Kisii, Kenya; d Nuffield Department of Medicine, University of Oxford, Oxford, UK; e Pwani University, Kilifi, Kenya; f Department of Psychiatry, University of Oxford, Oxford, UK

## Abstract

**Background:**

Neurological impairments might significantly contribute to reduced life expectancy in low-income and middle-income countries (LMICs). There are no empirical studies of premature mortality in children with neurological impairments in Africa. This study estimated the risk of premature mortality in children with neurological impairments and identified risk factors and causes of death.

**Methods:**

We did a cohort study based on a two-stage epidemiological survey in the Kilifi Health and Demographic Surveillance System (Kilifi, Kenya). Study participants were children aged 6–9 years. In the first stage, five trained field workers administered a low-cost screening tool to a random sample of households. In the second stage, we assessed for neurological impairments in five domains (epilepsy, cognitive impairments, vision impairments, hearing impairments, and motor impairments) using comprehensive clinical evaluation and extensive neuropsychological assessments. From the two-stage survey we identified a cohort of children with neurological impairment and a cohort of matched controls. We also enrolled an age-matched sample from the general population. The primary outcome was all-cause mortality. Mortality rates, standardised mortality ratio (SMR), and hazard ratios (HR) for risk factors were estimated and causes of death identified.

**Findings:**

We enrolled 306 children with neurological impairment, 9912 survey controls, and 22 873 age-matched participants from the general population, and followed up the cohorts between June 1, 2001, and Aug 31, 2018. Median follow-up was 14·5 years (IQR 8·6–17·2). 11 (3·9%) of 284 children with neurological impairment, 92 (1·0%) of 9009 controls, and 272 (1·2%) of 22 873 participants in the general population sample died during the follow-up. Overall mortality rates were 309·8 per 100 000 person-years of observation (95% CI 126·7–492·9) in children with neurological impairment, 80·8 per 100 000 person-years of observation (64·3–97·3) in controls, and 98·8 per 100 000 person-years of observation (87·1–110·6) in the general population sample (mortality rate ratio 3·83, 95% CI 2·05–7·16, p<0·001, compared with controls; 3·13, 1·71–5·72, p<0·001, compared with the general population). Mortality risk in children with neurological impairment was not dependent on the severity of impairment (p=0·291) nor on a specific neurological impairment domain (p=0·205). The overall risk of death adjusted for age and sex was higher in children with neurological impairment compared with controls (HR 4·24, 95% CI 2·26–7·94, p=0·002). An SMR of 3·15 (95% CI 1·66–5·49) was obtained after using the general population sample as the reference for indirect standardisation. In multivariable risk factor analysis, developmental delay (adjusted HR 18·92, 95% CI 2·23–160·44, p=0·007) and severe malnutrition (20·92, 3·14–139·11, p=0·002) increased the risk of mortality in children with neurological impairment. Infections such as HIV/AIDS and accidents were common among all decedents.

**Interpretation:**

The risk of premature mortality was higher in children diagnosed with neurological impairments compared with the general population and was increased by developmental delay and severe malnutrition. Child development and nutritional status should be assessed in all children in LMICs and tailored interventions started to improve outcomes.

**Funding:**

Wellcome Trust, DELTAS Africa Initiative.

## Introduction

There has been a significant global reduction in childhood mortality since 1990 due to a decline in infectious diseases, improved nutrition, and better outcomes of neonatal diseases. This decline differs by geographical region, with most deaths still occurring in low-income and middle-income countries (LMICs).^1^, ^2^, ^3^ Improved survival beyond age 5 years is likely to have resulted in a greater burden of neurological impairments and disability in older children.^4^ Children with neurological impairments are additionally exposed to infections and environmental factors that increase or modify the risk of premature mortality.^5^ However, the extent of this risk in older children with neurological impairments remains unknown in many LMICs.

Studies investigating mortality outcomes in people with epilepsy and intellectual disabilities suggest an increased risk of premature mortality compared with the general population.^6^ Other neurological impairment domains are also expected to increase mortality in children and adolescents: some studies have shown an association between mental disorders and other co-morbidities with mortality.^7^, ^8^ Empirical evidence about the risk of premature mortality in children with neurological impairments, associated risk factors, and causes of death is important in developing policies and interventions that prevent mortality in affected children.

**Research in context**

**Evidence before this study**

Mortality is increased in children with neurological impairments, but evidence is scarce in Africa, where studies have investigated single disorders such as epilepsy and intellectual disability. We searched PubMed (with MeSH) and Google Scholar for reports published between 1990 and 2018 in English with the search terms (“Excess Mortality OR Premature Mortality”) AND “Neurological Impairments” AND “Children”. Although there was evidence of increased risk of premature mortality in children with neurological impairments compared with the general population, particularly in children with epilepsy and intellectual disability, there were no single studies examining mortality in different neurological impairment domains. Risk factors associated with increased premature mortality were self-harm, accidents and injuries, cardiovascular diseases, respiratory diseases, cancer, and other medical comorbidities. Most of the neurological impairment studies originated from high-income countries (HICs) and there were no studies of mortality following different neurological impairment domains in low-income and middle-income countries (LMICs), where more deaths are expected.

**Added-value of this study**

306 children with neurological impairments and 9912 controls identified in a community-based survey were prospectively assessed from June 1, 2001, to Aug 31, 2018, and incident mortality rates compared with an age-matched sample from the general population. The risk of mortality was about three to four times higher in children with neurological impairment compared with controls and the age-matched sample from the general population. We also identified developmental delay and severe malnutrition as risk factors that increase the risk of premature mortality in children with neurological impairments.

**Implications of all the available evidence**

The findings provide evidence that neurological impairments, including domains not previously investigated such as deficits in cognitive and motor functions, are associated with premature mortality and these data might contribute to more precise estimates of burden in LMICs. Targeted interventions for neurodevelopmental delay and severe malnutrition are needed to improve survival in children with neurological impairment.

We hypothesised that the risk of premature mortality is higher in children with neurological impairments than in children without these disorders and in the general population. We estimated mortality rates in children diagnosed with neurological impairments compared with controls from a survey and a sample from the general population. We also sought to identify predictors of premature mortality in children with neurological impairments, and the putative causes of death among the decedents.

## Methods

### Study design and participants

We did a cohort study (based on a two-stage epidemiological survey) between June 1, 2001, and Aug 31, 2018, in the Kilifi Health and Demographic Surveillance System (KHDSS),^9^ a defined geographical area located along the Kenyan coast. Established in 2000, the KHDSS supports community-based surveillance of vital statistics such as births and deaths, which are integrated with hospital-based and field-based research systems. The main economical activity in this rural setting is subsistence farming, although some residents are fishermen and others benefit from tourism. The formal health-care system comprises a government referral hospital, peripheral dispensaries, and private facilities. Neonatal and under-5 mortality rates are 17·1 and 41·0 deaths per 1000 livebirths, respectively.^9^

The KHDSS covers an area of around 900 km^2^ with a population of approximately 280 000 residents under active surveillance. The age-structure is pyramid shaped and many of this population are children (around 49% are aged <15 years); the population size decreases evenly with increasing age. The male to female ratio is 88:100. Although this demographic structure is similar to most parts of Kenya, Kilifi county is one of the poorest counties, with about half of the population surviving on less than the equivalent of US $1 dollar per day.

Study participants were children aged 6–9 years.^10^ We focused on this age group rather than younger children because: (1) adaptation and validation of neuropsychological assessments are more reliable; (2) diagnosis of epilepsy is not confused with febrile seizures; (3) neurological sequelae of neonatal insults are more apparent at this age; and (4) school-going children are easier to target for public health interventions.

We obtained approval for the epidemiological study from the National Ethical Review Committee of the Kenya Medical Research Institute under protocol number 518, and permission to use data from the KHDSS was granted by the Data Governance Committee at the KEMRI Wellcome Trust Research Programme in Kilifi. Written informed consent was sought from the parents, guardians, or caretakers of the participants in all stages before data collection.

### Baseline epidemiological study

We estimated the prevalence and risk factors for neurological impairment in the participants in two stages.^10^ In the first stage, we used random sampling to select participating households for screening. All eligible children (6–9 years) and households were randomly identified from the KHDSS database. Five trained field workers administered a low-cost screening tool for detecting moderate to severe neurological impairment, the Ten Question Questionnaire,^11^ after obtaining consent. We addressed interobserver and intraobserver variability during the pre-study training and pilot stages.^11^ All children who screened positive and a random proportion of those (8·3%) who screened negative were invited for the second stage.

In the second stage, we assessed for neurological impairments in five domains (epilepsy, cognitive impairments, vision impairments, hearing impairments, and motor impairments) using comprehensive clinical evaluation and extensive neuropsychological assessments. The assessment team comprised three trained clinicians and five psychologists working under the supervision of a paediatric neurologist (CRJCN). A diagnosis of epilepsy was based on a history of seizures, and an electroencephalogram (EEG) was used to characterise and classify epilepsy. We defined cognitive impairments from Z scores obtained from standardised neuropsychological assessments.^10^ The Sonksen-Silver acuity system and calibrated audiometry were used to assess vision and hearing impairments, respectively. Assessment of motor impairments was by clinical examination using a standardised examination form.^10^ We obtained sociodemographic information and a history of child development using a structured parental questionnaire.^10^ Height and weight for each child were measured. A child with neurological impairment was defined as a child with a confirmed impairment in at least one of the five domains that were assessed, each classified by severity status as mild, moderate, or severe according to WHO criteria.^12^

Survey controls comprised those who did not have neurological impairments as assessed in the two stages of the study. We also enrolled an age-matched sample identified from the same demographic surveillance database from which the random selection was chosen—ie, a general population sample. This allowed computation of SMR using indirect standardisation.

### Follow-up of study participants and measurement of mortality

We followed up the children with neurological impairments, controls, and the general population sample from June 1, 2001, to Aug 31, 2018. Participants exited the risk pool at the end of the study, on their date of death, or on outmigration. 30 trained field workers visited participants' households once every 4–6 months during the follow-up to assess mortality and migration status using standardised data collection instruments. The primary outcome for this study was all-cause mortality as established during these visits. Person-years of observation for each participant were calculated and individuals who either migrated out of the KHDSS or were alive at the end of the follow-up were right-censored during the analysis.

Formal registration of mortality is poor in most LMICs because many deaths occur outside the formal health-care system.^14^ Verbal autopsy, an interview with a close relative of the deceased to identify symptoms and circumstances preceding death, is a recognised alternative where vital registration is missing or inadequate.^3^, ^15^, ^16^, ^17^ WHO provides a standard questionnaire for different age groups, and the information provided by the informant is analysed by a medical expert or a computer algorithm to assign the cause of death.^18^ Verbal autopsies have been done in the KHDSS since 2008.^19^ If a participant died before 2008, we could not determine the causes of death. For this study, either the guardian or the carer of the deceased individual before death was interviewed within 1–4 months after the participant's death to allow for a culturally acceptable period of mourning. Putative causes of death were assigned according to the 10th version of the International Classification of Diseases (ICD-10) using the InterVA-4 computer-based probabilistic model, as in previous studies.^9^, ^19^

### Statistical analysis

Assuming a mortality rate of 9·1% and 0·5% in children with and without neurological impairments, respectively,^13^ a 17-year cohort study required at least 77 cases of neurological impairment and controls each to yield reliable estimates of the risk of mortality with 80% power at 95% significance level. Overall, cause-specific, and sex-adjusted and age-adjusted mortality rates were computed for the children with neurological impairments, controls, and the general population sample. Mortality rate ratios (MRR) and their 95% CI were used to compare mortality rates. We did indirect standardisation using mortality rates from the general population sample as the reference to compute the standardised mortality ratio (SMR). Sensitivity analysis involved weighting our findings according to the SMR and follow-up duration from a previous systematic review of premature mortality in epilepsy.^13^ Cox proportional hazard regression models were fitted using R packages for survival analysis^20^, ^21^ to compute hazard ratios (HR) and investigate the contribution of age, sex, nutritional status, developmental history, and other risk factors on mortality among children with neurological impairments. Nutritional status was measured by weight-for-age, height-for-age, and weight-for-height *Z* scores using WHO's child growth charts (2007) as the reference. Severe malnutrition was defined as a child with a *Z* score of less than −3 in either of the three measures. We checked the proportional hazards assumption and assessed the appropriate functional forms for continuous variables in the final model. Proportionate mortalities by cause were estimated for deaths occurring after 2008 based on verbal autopsy reports. Statistical analyses were done in R software environment for statistical computing and graphics.^22^

### Role of the funding source

The funders of the study had no role in the study design, data collection, data analysis, data interpretation, or writing of the report. The authors had full access to all the data in the study and had final responsibility for the decision to submit for publication.

## Results

**Figure 1:**
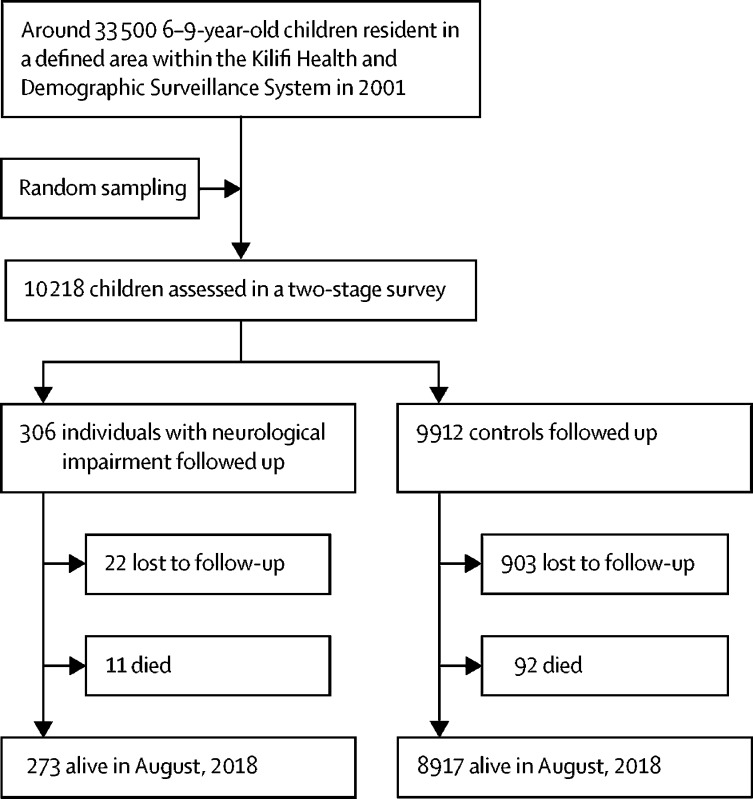
Flow diagram showing recruitment and follow-up of individuals with neurological impairments and controls

In the baseline survey, 10 218 children were screened in the first stage (figure 1). A cohort of 306 children with neurological impairments and 9912 controls were identified from the two-stage survey for follow-up. There were 22 873 participants in the general population sample.

The median duration of follow-up was 14·7 years (IQR 9·2–16·8), 14·8 years (9·6–16·7), and 14·0 (8·0–17·2) years for the children with neurological impairment, controls, and the general population sample, respectively. Most of the children with neurological impairment (284 [92·8%] of 306) and controls (9009 [90·0%] of 9912) completed the follow-up study. Participants lost to follow-up were similar to those completing the study in terms of age and sex; those lost to follow-up were excluded from the main analyses. Further details about the cases, controls, and the general population sample are in the appendix p 1.

11 (3·9%) of 284 children with neurological impairment, 92 (1·0%) of 9009 controls, and 272 (1·2%) of 22 873 participants in the general population sample died during follow-up. A total of 375 deaths occurred in 392 581 person-observation years, yielding a crude mortality rate of 95·5 per 100 000 person-observation years (95% CI 85·9–105·2). Overall mortality rates were 309·8 per 100 000 person-observation years (126·7–492·9) in children with neurological impairment, 80·8 per 100 000 person-observation years (64·3–97·3) in controls, and 98·8 per 100 000 person-observation years (87·1–110·6) in the general population sample. The risk of mortality in children with neurological impairment was greater than the risk in controls (MRR 3·83, 95% CI 2·05–7·16, p<0·001) and the general population sample (3·13, 1·71–5·72, p<0·001).

The risk of mortality in children with neurological impairment was neither affected by the severity of impairment nor by individual neurological impairment domain (table 1). MRR was 1·02 (95% CI 0·17–6·13, p=0·979) when mortality in participants with severe neurological impairment was compared with moderate impairment. The ratio was 2·01 (95% CI 0·41–9·94, p=0·384) when mortality in participants with severe neurological impairment was compared with mild impairment. Similarly, mortality in participants with moderate neurological impairment was similar to mortality in those with mild impairment (1·96, 95% CI 0·49–7·84, p=0.33). No individual domain of neurological impairment was associated with an increased risk of premature mortality compared with the rest (table 1).

**Table 1:** Mortality rates grouped by severity of neurological impairments and individual domain of impairment

	**Deaths (n=11)**	**Person-years of observation**	**Mortality rate per 100 000 person-years of observation (95% CI)**	**p value**
Severity level	..	..	..	0·291
Severe	2 (18%)	414·8	482·1 (0–1150·3)	..
Moderate	3 (27%)	637·1	470·9 (0–1003·7)	..
Mild	6 (55%)	2498·6	240·1 (48·0–432·3)	..
Domain*	..	..	..	0·205
Motor impairments	3 (27%)	571·7	524·8 (0–1118·6)	..
Cognitive impairment	9 (82%)	1803·9	498·9 (173·0–824·9)	..
Epilepsy	3 (27%)	1189·0	252·3 (0–537·8)	..
Hearing impairment	2 (18%)	860·1	232·5 (0–554·8)	..
Visual impairment	0	201·9	0	NA

* Domains of neurological impairment were not mutually exclusive and some individuals who died had more than one impairment. p value is for comparison of four domains.

**Figure 2:**
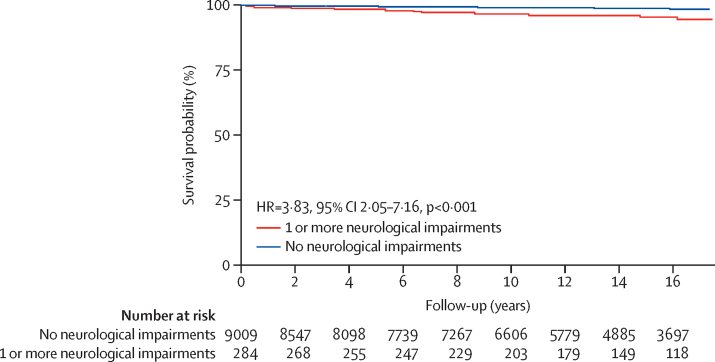
Estimated survival probability comparing individuals with neurological impairment versus controls

The probability of survival in children with neurological impairment was significantly lower than in controls (log-rank p<0·001; figure 2). The overall risk of mortality was four times higher in children with neurological impairment than in controls (figure 2), and the risk of mortality increased by about 11% after adjusting for age and sex (adjusted HR 4·24, 95% CI 2·26–7·94, p=0·002). The model adjusted for epilepsy changed the HR by 9·0% which was less than the model adjusted for cognitive impairment (65·8% change), motor impairment (14·4% change), and hearing impairment (12·0% change).

Age (p=0·232) and sex (p=0·821) differences did not affect the risk of mortality in children with neurological impairments or the general population sample (table 2). The SMR in children with neurological impairments was 3·15 (95% CI 1·66–5·49). An SMR of 3·89 (1·94–10·75) was obtained after sensitivity analysis.

**Table 2:** Age-specific and sex-specific mortality rates

		**Neurological impairment cases**			**General population sample**		
		Deaths (n)	Person-years of observation	Mortality rate per 100 000 person-years of observation (95% CI)	Deaths (n)	Person-years of observation	Mortality rate per 100 000 person-years of observation (95% CI)
Age of participant at the start of the study							
	6 years	3	1424	210·6 (0–449·9)	67	71642	93·5 (71·1–115·9)
	7 years	1	825	121·2 (0–358·7)	73	70946	102·9 (79·3–126·5)
	8 years	4	585	684·3 (13·7–1354·9)	65	67802	95·9 (72·6–119·7)
	9 years	3	717	418·6 (0–892·4)	67	64794	103·4 (78·7–128·2)
Male		5	1825	274·0 (33·8–514·1)	149	141 817	105·1 (88·2–121·9)
Female		6	1726	347·6 (69·5–625·9)	123	133 366	92·2 (75·9–108·5)
Total		11	3551	309·8 (126·7–492·9)	272	275 184	98·8 (87·1–110·6)

Data are for individuals with neurological impairments versus the general population sample.

Developmental delay characterised by the inability to self-feed at age 2 years, inability to assist in household chores, and severe malnutrition were associated with an increased risk of mortality in children with neurological impairment at the univariable level (table 3). Severe malnutrition and an inability to self-feed at age 2 years remained associated with increased risk of mortality in the final multivariable model.

**Table 3:** Sociodemographic, medical and developmental history and their association with mortality in children with neurological impairments*

		**Univariable HR (95% CI)**	**p value**	**Multivariable HR (95% CI)**	**p value**
Age of participants at the start of the cohort study					
	6 years	1·00	..	1·00	..
	7 years	0·57 (0·06–5·49)	0·627	0·51 (0·04–7·15)	0·621
	8 years	3·21 (0·72–14·38)	0·127	6·94 (1·14–42·95)	0·037
	9 years	1·97 (0·40–9·78)	0·407	4·14 (0·62–27·70)	0·142
Male participant		0·78 (0·24–2·55)	0·678	0·92 (0·24–3·51)	0·905
A father without an income–generating activity		0·63 (0·13–3·03)	0·563	..	..
A mother without an income–generating activity		0·81 (0·23–2·89)	0·751	..	..
Severe malnutrition		16·74 (3·47–80·74)	<0·001	20·92 (3·14–139·11)	0·002
BMI (kg/m^2^)		0·56 (0·29–1·08)	0·083	..	..
Baby did not cry immediately after delivery		1·10 (0·14–8·61)	0·929	..	..
Abnormal pregnancy		1·46 (0·68–5·51)	0·577	..	..
Could not walk at age 2 years		2·10 (0·61–7·16)	0·238	0·72 (0·13–3·89)	0·698
Could not talk at age 2 years		1·16 (0·34–3·98)	0·807	..	..
Could not self–feed at age 2 years		4·41 (1·29–15·08)	0·018	18·92 (2·23–160·44)	0·007
Could not pick up objects at age 2 years		3·47 (0·44–27·17)	0·236	0·45 (0·03–8·24)	0·591
Inability to meet urination or defecation needs at age 6–9 years		4·21 (0·91–19·5)	0·066	0·78 (0·05–11·88)	0·855
Inability to assist in household chores between age 6–9 years)		5·85 (1·55–22·12)	0·009	4·12 (0·36–47·02)	0·254
Inability to self–clean at age 6–9 years		3·27 (0·71–15·17)	0·129	2·92 (0·27–31·56)	0·376

HR=hazard ratio. BMI=body-mass index.

* n=284.

Verbal autopsy reports were available for five (45·5%) of 11 deaths in children with neurological impairments, 39 (42·4%) of 92 deaths in controls, and 100 (36·8%) of 272 deaths in the general population sample. Three (27·3%) of 11 deaths in children with neurological impairment were HIV/AIDS-related, one caused by an accident, and another resulting from an assault. HIV/AIDS was related to 26 (18·1%) of 144 deaths whose putative causes were determined. Accidents, including road or traffic accidents and drowning, caused 23 deaths (16·0%) and neurological conditions such as epilepsy, meningitis, and encephalitis caused 12 (8·3%) deaths. Further details about cause-specific mortality from verbal autopsy reports are in the appendix (p 2).

## Discussion

Mortality in children with neurological impairments in this population from rural Kenya was three to four times higher than in controls and a sample from the general population. The higher mortality rates in children with neurological impairment compared with controls were consistent with the Cox proportional hazards model which showed that more children without neurological impairment were surviving than those with neurological impairment. The result is also consistent with those from studies of individual neurological impairments such as epilepsy.^23^

These mortality rates are probably a conservative minimum since the sensitivity analysis showed underestimation by about 24%. Deaths are a culturally sensitive issue in this community, and many might have gone unreported. People obtain permits of burials from local chiefs and so there are usually no death certificates for verification. Additionally, this study did not include younger age groups (<6 years) which might have had greater mortality. The risk of death in children with severe impairments is significantly higher during the first 5 years of life than the succeeding years^24^ and will be missed by studies following up children older than 5 years. Although more focus in neurological impairment is usually given to morbidity, quality of life, and wellbeing,^25^ our results suggest there is a need to get concerned about the risk of death in survival cohorts.

Few studies have examined the mortality rate in children with different domains of neurological impairment worldwide; most report only proportional mortality for single neurological impairment conditions such as epilepsy, sometimes without comparison with the general population.^26^ An epilepsy clinic was established for a previous study,^23^ which might partly account for the differences in mortality estimates compared with this study. A review^13^ of premature mortality in epilepsy reported a median SMR of 2·6 (95% CI 1·3–7·2), although the duration of follow-up from the pooled studies was shorter than in our study (mean 8·3 years). This finding suggests that other non-epilepsy conditions such as intellectual disability might have as serious consequences as epilepsy. Our study followed up children with neurological impairments and those from the general population sample for mortality using the same methods, unlike another study^23^ in which children with neurological impairment were followed up more frequently and keenly than those without neurological impairment in the general population. We found that mortality rates did not vary according to individual domains of neurological impairment. However, a Cox regression model adjusted for epilepsy changed the HR by only 9%, which is far less than the model adjusted for cognitive impairment (66% change), motor impairment (14% change), and hearing impairment (12% change). These findings point to the significance of cognitive and motor impairments on premature mortality in this setting. Children with these impairments are likely to have cerebral palsy for which premature mortality is widely recognised.^24^ The probability of childhood survival to adulthood in children with cerebral palsy is significantly reduced by comorbid impairments of motor, cognitive, or visual functions.^27^ Although no deaths were reported in children with vision impairment, the global burden is enormous^28^ and future studies should assess associated disability-adjusted life years.

Surprisingly, mortality rates for participants with severe neurological impairment were similar to rates for those with moderate and mild neurological impairment. Mortality rates in those with neurological impairment also did not differ according to age and sex, which suggests these groups were equally vulnerable to mortality. Although there were few age bands, this finding was replicated in the general population sample. A different study that used a similar study population^29^ suggested that male individuals die earlier because their adventurous nature exposes them to accidents and falls, but other genetic and hormonal differences should be examined. A study also showed that more deaths by suicide occur in male individuals than females in this rural area.^7^ Previous population-based studies document that more males might die,^29^ but there are few studies reporting mortality in different neurological impairment domains, which complicates comparisons with our study.

The risk of death in children with neurological impairment was increased by developmental delay and severe malnutrition. Developmental delay is a proxy for psychomotor development,^30^ implying that mortality rates are greater in children with neurological impairment. Severe malnutrition increased the risk of mortality, which is not surprising since malnutrition is already known to cause premature mortality in children.^31^ Severe malnutrition might also be a measure of compromised immunity,^32^ suggesting that malnourished children would be susceptible to other childhood infections that can cause death. These results highlight the importance of assessment of developmental and nutritional status in children with neurological impairments as these might help to identify those at risk of premature mortality and development of targeted interventions. Our findings for malnutrition and developmental delay, however, should be interpreted cautiously because these factors were measured at the baseline and we did not investigate whether recovery from developmental delay and malnutrition during the follow-up affected mortality.

Cause of death could only be established in fewer than half the individuals followed up, but it was clear that infections were related to mortality in children with neurological impairments. HIV/AIDS was common in most of the deaths occurring in the neurological impairment cohort, and the finding was consistent in both comparison groups.^33^ Although HIV has been identified as an important cause of neurodevelopmental problems, these findings suggest that the acquisition of the infection in children who already had neurological impairment might change the course of neurodisability including increased mortality. Public health interventions to reduce infections in children should be sustained. Assaults and accidents were also common causes of death, in both individuals with and without neurological impairment. This study identified that epilepsy and meningitis were related to mortality in Africa, supporting previous studies.^13^, ^34^

Limitations of our study included the small number of observations of death, especially in the neurological impairment cohort; that risk factors were measured at baseline and it is difficult to determine if they changed during the study and whether this affected the results; that the causes of death were only available for part of the study period, which is when the verbal autopsy was introduced in the demographic surveillance system, and there were no coroner reports; and that this study was done in a single geographical area in rural Kenya, which might limit the generalisability of the findings to other areas in Kenya and other LMICs. This study, however, had unique strengths such as having two comparison groups and robust statistical analyses.

In conclusion, to the best of our knowledge, this study provides the first evidence in LMICs that mortality is about three to four times greater in children with different neurological impairments than in controls and the general population. The risk of mortality in children with neurological impairments was increased by developmental delay and severe malnutrition. Child development and nutritional status should be assessed and addressed in all children presenting with neurological impairments in LMICs.

## Data sharing

The data that support the findings of this study are available from the KEMRI-Wellcome Trust Research Programme but restrictions apply to the availability of these data, according to the consenting process during the study. Those interested in the data can apply through the data governance committee of the KEMRI-Wellcome Trust Programme at https://kemri-wellcome.org/about-us/.
