# Supplementary material for: Premature mortality in children aged 6–9 years with neurological impairments in rural Kenya: a cohort study
Source: Lancet Glob Health. 2019 Oct 22;7(12):e1728–35. doi: 10.1016/S2214-109X(19)30425-5 (PMC7024990; doi:10.1016/S2214-109X(19)30425-5)
Supplement: Supplementary appendix [file mmc1.pdf]

THE LANCET
Global Health

Supplementary appendix

This appendix formed part of the original submission and has been peer reviewed. We post it as supplied by the authors.

Supplement to: Abuga JA, Kariuki SM, Kinyanjui SM, Van Hensbroek MB, Newton CRJC. Premature mortality in children aged 6–9 years with neurological impairments in rural Kenya: a cohort study. *Lancet Glob Health* 2019; published online Oct 22. http://dx.doi.org/10.1016/S2214-109X(19)30425-5.

Supplementary Material

Supplementary Table 1: A description of the cases, controls and general population sample of the cohort study conducted in Kilifi, Kenya

Cohort	Neurological impairment cases		Controls		General population
**Factor**	**[b]Completed N=284**	**LFU N=22**	**[b]Completed N=9009**	**LFU N=903**	**Completed N=22, 873**
[a]Age					
6 years	106 (37·3%)	9 (40·9%)	2570 (28·5%)	255 (28·2%)	5, 801 (25·4%)
7 years	65 (22·9%)	6 (27·3%)	2198 (24·4%)	224 (24·8%)	5, 787 (25·3%)
8 years	53 (18·7%)	4 (18·2%)	2114 (23·5%)	220 (24·4%)	5, 713 (25·0%)
9 years	60 (21·1%)	3 (13·6%)	2127 (23·6%)	204 (22·6%)	5, 572 (24·4%)
Sex					
Female	139 (48·9%)	8 (36·4%)	4472 (49·6%)	439 (48·6%)	11, 329 (49·5%)
Male	145 (51·1%)	14 (63·6%)	4537 (50·4%)	464 (51·4%)	11, 544 (50·5%)
School attendance					
No	171 (60·2%)	10 (45·5%)	3612 (40·1%)	358 (39·6%)	
Yes	113 (39·8%)	12 (54·5%)	5397 (59·9%)	545 (60·4%)	
Person years of follow-up (PYO)	3, 551	-	113, 846	-	275, 184
Mean years of follow-up (SD)	12·5 (5·1)		12.6 (5.0)		12·0 (5·6)
Median years of follow-up (IQR)	14·7 (9·2-16·8)	-	14·8 (9·6-16·7)	-	14·0 (8·0-17·2)

Abbreviations: LFU = loss to follow-up; PYO = person-years of observation; SD = standard deviation; IQR = inter-quartile range.
[a]Age of participant at the start of the cohort study.
[b]There was no significant difference between participants completing the study and those lost to follow-up among NI cases (age p-value = 0·831, sex p-value = 0·276, and school attendance p-value = 0·175) and controls (age p-value = 0·868, sex p-value = 0·558, and school attendance p-value = 0·794).

Supplementary Table 2: Cause-specific mortality from verbal autopsy reports for participants dying between 2008-2018

Cause of death (ICD-10 Classification)	NI cases N (%)	Controls N (%)	General population N (%)	All deaths N (%)
HIV/AIDS-related	3 (27·3)	6 (6·5)	17 (6·3)	26 (6·9)
Accidents (road/traffic, drowning and falls)	1 (9·1)	5 (5·4)	17 (6·3)	23 (6·1)
Epilepsy, Meningitis and encephalitis	0	3 (3·3)	9 (3·3)	12 (3·2)
Malaria	0	3 (3·3)	6 (2·2)	9 (2·4)
Acute respiratory infections including pneumonia	0	5 (5·4)	5 (18)	10 (2·7)
Any specified or unspecified neoplasm	0	2 (2·2)	7 (26)	9 (2·4)
Pregnancy related deaths	0	2 (2·2)	5 (1·8)	7 (1·9)
Assault	1 (9·1)	2 (2·2)	6 (2·2)	9 (2·4)
Pulmonary Tuberculosis	0	1 (1·1)	3 (1·1)	4 (1·1)
Cardiac disease: acute or unspecified	0	2 (2·2)	4 (1·5)	6 (1·6)
Intentional self-harm	0	1 (1·1)	4 (1·5)	5 (1·3)
Acute abdomen	0	0	1 (0·4)	1 (0·3)
Stroke	0	0	2 (0·7)	2 (0·5)
Renal failure	0	0	2 (0·7)	2 (0·5)
Liver cirrhosis	0	0	1 (0·4)	1 (0·3)
Severe Malnutrition	0	1 (1·1)	2 (0·7)	3 (0·8)
Other unspecified NCD	0	1 (1·1)	1 (0·4)	2 (0·5)
Other unspecified infectious diseases	0	1 (1·1)	1 (0·4)	2 (0·5)
Diarrheal diseases	0	0	1 (0·4)	1 (0·3)
Contact with venomous plants/animals	0	0	1 (0·4)	1 (0·3)
Sickle cell with crisis	0	1 (1·1)	1 (0·4)	2 (0·5)
Haemorrhagic fever	0	1 (1·1)	1 (0·4)	2 (0·5)
Diabetes Mellitus	0	1 (1·1)	1 (0·4)	2 (0·5)
COPD	0	1 (1·1)	1 (0·4)	2 (0·5)
Asthma	0	0	1 (0·4)	1 (0·3)
[a]Unclassified deaths	6 (54·5)	53 (57·6)	172 (63·2)	231 (61·6)
**TOTAL**	11	92	272	375

Abbreviations: ICD = international classification of disease; NI = neurological impairment; NCD = non-communicable diseases; COPD = chronic obstructive pulmonary disease.
[a]Unclassified deaths from 2001-2008 because verbal autopsies were not conducted.
